# Heavy Tailed Distributions of Effect Sizes in Systematic Reviews of Complex Interventions

**DOI:** 10.1371/journal.pone.0034222

**Published:** 2012-03-27

**Authors:** Christopher Burton

**Affiliations:** Centre for Population Health Sciences, University of Edinburgh, Edinburgh, United Kingdom; Cuban Neuroscience Center, Cuba

## Abstract

**Background:**

Systematic reviews of complex interventions commonly find heterogeneity of effect sizes among similar interventions which cannot be explained. Commentators have suggested that complex interventions should be viewed as interventions in complex systems. We hypothesised that if this is the case, the distribution of effect sizes from complex interventions should be heavy tailed, as in other complex systems. Thus, apparent heterogeneity may be a feature of the complex systems in which such interventions operate.

**Methodology/Principal Findings:**

We specified three levels of complexity and identified systematic reviews which reported effect sizes of healthcare interventions at two of these levels (interventions to change professional practice and personal interventions to help smoking cessation). These were compared with each other and with simulated data representing the lowest level of complexity. Effect size data were rescaled across reviews at each level using log-normal parameters and pooled. Distributions were plotted and fitted against the inverse power law (Pareto) and stretched exponential (Weibull) distributions, heavy tailed distributions which are commonly reported in the literature, using maximum likelihood fitting. The dataset included 155 studies of interventions to change practice and 98 studies of helping smoking cessation. Both distributions showed a heavy tailed distribution which fitted best to the inverse power law for practice interventions (exponent = 3.9, loglikelihood = −35.3) and to the stretched exponential for smoking cessation (loglikelihood = −75.2). Bootstrap sensitivity analysis to adjust for possible publication bias against weak results did not diminish the goodness of fit.

**Conclusions/Significance:**

The distribution of effect sizes from complex interventions includes heavy tails as typically seen in both theoretical and empirical complex systems. This is in keeping with the idea of complex interventions as interventions in complex systems.

## Introduction

Many interventions in health and social care are complex, in that they involve multiple interacting components [1] and are delivered in differing ways and circumstances [2]. These “complex interventions” contrast with more simple interventions such as a drug given to treat a single condition where most sources of variability can be identified and controlled for, either directly or by randomisation. Reviews of the effects of complex interventions, such as actions to change clinical practice, have shown over many years that effects are commonly small [3] and this has been attributed to various phenomena, most recently the complexity of healthcare systems [4].

**Figure 1:**
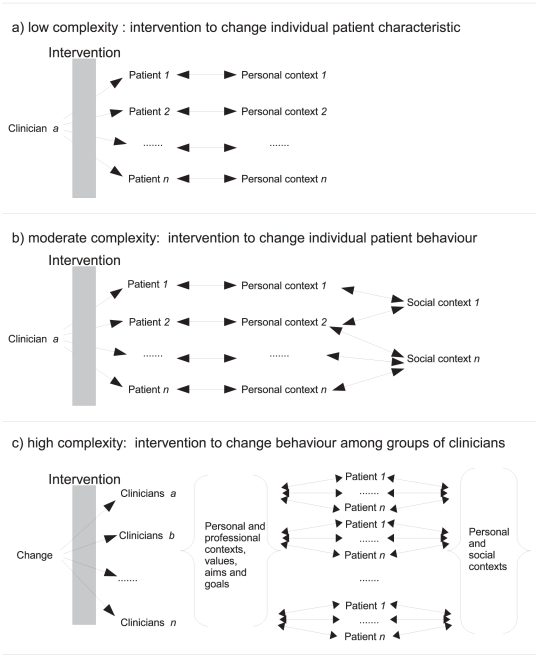
Schematic representation of three levels of complexity in relation to healthcare interventions. (a) shows a simple intervention given to individual and independent patients (for instance administration of a drug). (b) shows a moderately complex intervention – for example advice or support to help smoking cessation – where the treatment is delivered to individual patients but their networks of interaction – some of which may be shared – influence the outcome of the intervention. (c) shows a highly complex intervention – for example interventions to change clinical practice – where the intervention attempts to change the practice in order to deliver individual patient treatment. The effect of the intervention depends on interaction networks at the practice/clinician and at the patient level.

The possible link between complex interventions and the science of complex systems [5] has been elaborated by a number of authors [6]–[9]. They argue that complex interventions typically possess “sensitive” causality in which outcomes depend on multiple steps and interactions [6], although few published studies of complex interventions explicitly describe and model the complexity of the system they are studying [10], [11]. Figure 1 outlines three scenarios which display increasing complexity. In the first, the intervention applies to individuals (each with their own personal characteristics) in isolation; in the second the effect of the intervention depends both on the intervention and the environment with which individuals interact. In the third level, the intervention is applied to a healthcare team which then interacts with individuals who are in turn embedded in their own social networks. In the first level, with low complexity, variation within a population can be assumed to be due to statistical chance as each individual is independent. The second level, with moderate complexity can be understood using social cognitive theories such as the Theory of Planned Behaviour [12] which includes both personal elements such as intention and social effects such as norms. The third, high complexity level, extends the previous models by including a range of complex interactions affecting the healthcare system (whether individual, clinical team or whole system) which precede the delivery of care to patients. This extends the personal components of the Theory of Planned Behaviour with group ethos, aims and threats [13]–[15].

While to date the argument about whether complex interventions should be understood as interventions within complex systems has been largely philosophical, there are testable properties of complex systems [5], [16] which should be detectable in the results of complex interventions. One such property is the presence of characteristic heavy-tailed statistical distributions such as the inverse power law [17] and stretched exponential [18]. Such distributions, which appear to be ubiquitous in nature[17], [19] and have been found in healthcare systems [20], are very different from the normal distribution which characterises the distribution of simple effects. In particular, such distributions contain many more small values than a normal distribution, but also a few more extreme values.

We hypothesised that if complex interventions are “interventions in complex systems” [7] the effect sizes of these interventions should show a heavy-tailed distribution typical of those seen in other complex systems.

## Methods

### Objective

We examined the distribution of effect sizes reported within a series of systematic reviews of complex interventions to change practice. We then compared this with two control distributions: (i) effect sizes from systematic reviews of patient level interventions to stop smoking, which we took to represent moderate complexity as shown in figure 1, and (ii) simulated data representing random variation around a mean effect size.

### Selection of studies

In order to test the distribution of effects in complex healthcare systems we sought systematic reviews of interventions which (a) represented changes in systems (for instance the behavior of health care professionals) rather than to a single pathway (for instance a public health measure to add nutritional supplements to food) (b) had a range of possible responses (ranging from ignore, through minor change, to radical revision of a process of care), (c) had causal models with multiple stages in which changes were also likely to lead to trade-offs. The essence of these criteria was that we viewed practitioners as agents within systems with complex causal models and trade-offs between different actions. We chose to study interventions to change practitioner behaviour (either individually or in groups) from reviews published by the Cochrane EPOC collaboration. We selected this source because the process of conducting these reviews identifies and, where possible, quantifies a wide range of biases such that only methodologically robust studies are included.

We reviewed the list of all reviews published by October 2010 to identify those which (a) aimed to change physician behaviour (b) acted remotely from the clinical consultation, (c) included comparisons of at least 10 included studies, and (d) permitted extraction of individual study effect sizes. Criteria (a) and (b) were chosen to reflect the requirements for complexity; criteria (c) and (d) were chosen to permit consistent data reporting and analysis. We identified three reviews: audit and feedback as methods to change physician behaviour [21], educational outreach visiting [22] and continuing education meetings and workshops [23].

### Selection of control data

Smoking cessation data were collected from 4 systematic reviews in the Cochrane Database of Systematic Reviews Tobacco Control section. These examined the effect sizes from randomized controlled trials of the following smoking cessation strategies: Nicotine Replacement Therapy [24], physician advice [25], individual behavioural counseling [26] and motivational interviewing [27]. These were chosen to represent moderate complexity because while the treatment was delivered consistently, individual response would be likely to be at least partly socially determined.

Simulation data for independent samples comprised 10,000 points designed to represent a population of risk ratios. As the logarithm of the relative risk ratio is approximately normally distributed, we generated a random lognormal distribution with log-mean and log-standard deviation taken from the log transformed effect sizes for EPOC data.

### Extraction of data

For each review we selected all comparisons with more than 10 studies. We then extracted a measure of effect size from each study as follows: for comparisons with dichotomous outcomes, we used the relative risk adjusted for baseline differences. For comparisons reporting continuous outcomes we converted the value reported in the reviews – the proportional change in the intervention group relative to control mean and adjusted for baseline difference - and converted this to a relative risk ratio (relative risk ratio = 1+ adjusted proportional change). Where the aim of an intervention was a reduction in behaviour (e.g. reducing error) the effect was reversed such that in all cases a relative risk ratio greater than one indicated the desired outcome. Within each comparison, these measures were rescaled by transforming the values into natural logarithms, calculating a z score for each study using the log-mean and log-standard deviation for each comparison, then converting the z score back to risk ratios using the overall log-mean and log-standard deviation of the whole population. These data were then pooled so that the analysis was carried out on three datasets: pooled reviews to change practice; pooled reviews of smoking cessation therapy; and simulated data representing a comparable lognormal relative risk ratio population.

### Fitting of distributions

We chose to fit the data to two specific distributions, the inverse power law and the stretched exponential. The inverse power law (or Pareto) distribution has historically been associated with the behaviour of complex systems [19] although it has been argued that it may represent a special case, restricted to only a limited range of data, and that the use of an alternative – such as the stretched exponential (or Weibull) distribution, is more appropriate [18]. We considered fitting additional heavy tailed distributions, however given the relatively small numbers of studies in the review we wished to avoid the risks of over-specification and confined the analysis to the two listed above.

The distribution of pooled relative risks was first plotted as a histogram on conventional axes and then as a cumulative distribution on logarithmic axes. Plotting an inverse power law distribution this way would produce a straight line with negative slope equivalent to the power law exponent.

The pooled rescaled effect size distribution was then fitted to both the inverse power law (or Pareto) and stretched exponential (Weibull) distribution using maximum likelihood estimation (with maximization of the tail conditional loglikelihood for the Weibull fitting) as described by Clauset [18]. All distributions were fitted with a lower threshold of 1. Goodness of fit was reported as the log-likelihood and compared between distributions using the non-nested Vuong test. All analyses were carried out using published [18] scripts in R 2.14.

While the estimation of the usefulness of a healthcare intervention requires both size and direction (conventionally expressed as positive effects leading to better outcomes and negative effects to worse), the influence of system complexity on the distribution of effect sizes should be independent of direction. In view of this we used two approaches to deal with negative effects (ie relative risk less <1) prior to fitting distributions: (1) setting a threshold of 1, thereby effectively excluding negative studies; (2) calculating an “absolute” value by inverting all relative risks <1. Analysis was repeated for each of these conditions.

### Sensitivity analysis

One possible explanation for a skewed distribution of effect sizes in a systematic review is publication bias [28], whereby unexpectedly strong results are selectively published and, equally importantly, unremarkable weak results are not. Because our model of heavy tailed distributions from complex systems depends on most responses being small, if publication bias existed, small effect studies would tend to be under-reported. We did not attempt to assess whether publication bias was present, rather we considered what effect publication bias – if present – would have on the data. To do this we simulated the effect of publication bias using a bootstrapping procedure. This increased the number of small effect size studies by selectively resampling with replacement from studies in the pooled distribution whose rescaled effect size was below the median value. These resampled studies were added to the original data to increase the size of the dataset by up to 80 points in order to simulate up to one third of all studies being unpublished because of small absolute effects. This bootstrapping procedure was repeated 200 times. The results of this process were plotted to show the effect of adding bootstrapped studies to the original data on the parameters and log-likelihoods of the model fit for the stretched exponential and inverse power law (using the same thresholds as previously).

### Ethics

This study comprised a secondary analysis of published data, no ethical permissions were required.

## Results

### Data from comparisons

There were 55 current systematic reviews in the Cochrane EPOC collection available for inspection at the start of the analysis. 16 of these related to changing practitioner behaviour of which 9 contained more than 10 studies. Four of these related to a range of approaches of addressing specific problems (for instance antibiotic prescribing) while five related to approaches (such as audit and feedback) across problems. Of these, two (audit and feedback [21] and educational outreach visiting [22]) had publicly available detailed data available [29]. Similar tables for a third review [23] were obtained from the authors. These three reviews contained 6 eligible comparisons with more than 10 studies and reported 166 outcomes. For 11 of these there was no measure of change adjusted for baseline and these were discarded leaving 155 outcomes which represented the dataset for this analysis. 72 outcomes were drawn from the review of audit and feedback, 51 from educational outreach visiting and 32 from continuing education meetings. Outcomes were continuous for 31 and dichotomous for 124. Twelve study outcomes appeared in two comparisons, two with continuous and dichotomous measures for the same study and ten appearing in two reviews (for example a study which included audit and feedback with educational outreach visiting could feature in both reviews). There were 54 systematic reviews in the Cochrane Tobacco Addiction Group database from which we identified the four individual reviews with more that 10 studies more comparison [24]–[27]. The number of outcomes in each comparisons, and a summary of the rescaled effect sizes drawn from the reviews are shown in table 1.

**Table 1:** Characteristics of each comparison included in the analysis. Values represent rescaled effect sizes within each comparison.

Review	Comparison	Continuous or dichotomous	N	Median	Interquartile range	Minimum	Maximum
**A. Interventions to change practice**							
Audit & Feedback [21]	Audit & Feedback alone	C	13	1.22	1.12 to 1.68	1.05	1.99
	Audit & Feedback alone	D	25	1.07	0.98 to 1.18	0.71	2.16
	Multifaceted including audit & feedback	D	34	1.10	1.03 to 1.36	0.78	18.3
Educational outreach visits [22]	Any intervention including educational outreach visits	C	18	1.22	1.12 to 1.41	1.00	7.17
	Any intervention including educational outreach visits	D	33	1.11	1.07 to 1.35	0.78	4.25
Continuing education meetings & workshops [23]	CME –professional outcomes	C	32	1.32	1.07 to 1.90	1.00	4.57
Combined rescaled data			155	1.16	1.05 to 1.49	0.64	8.17
**B. Interventions for smoking cessation**							
Nicotine replacement therapy [24]	Any NRT vs placebo/no NRT	D	50	1.34	1.19 to 1.98	0.50	4.33
Counselling [26]	Counselling vs control	D	17	1.56	1.32 to 2.01	0.58	5.5
Physician advice [25]	Minimal intervention	D	17	1.58	1.03 to 2.28	0.95	4.56
Motivational interviewing [27]	Motivational interviewing	D	14	1.62	1.16 to 1.99	0.92	5.28
Combined rescaled data			98	1.47	1.14 to 2.08	0.47	5.62
**C. Simulated data**		D	10000	1.31	1.02 to 1.69	0.33	4.78

**Figure 2:**
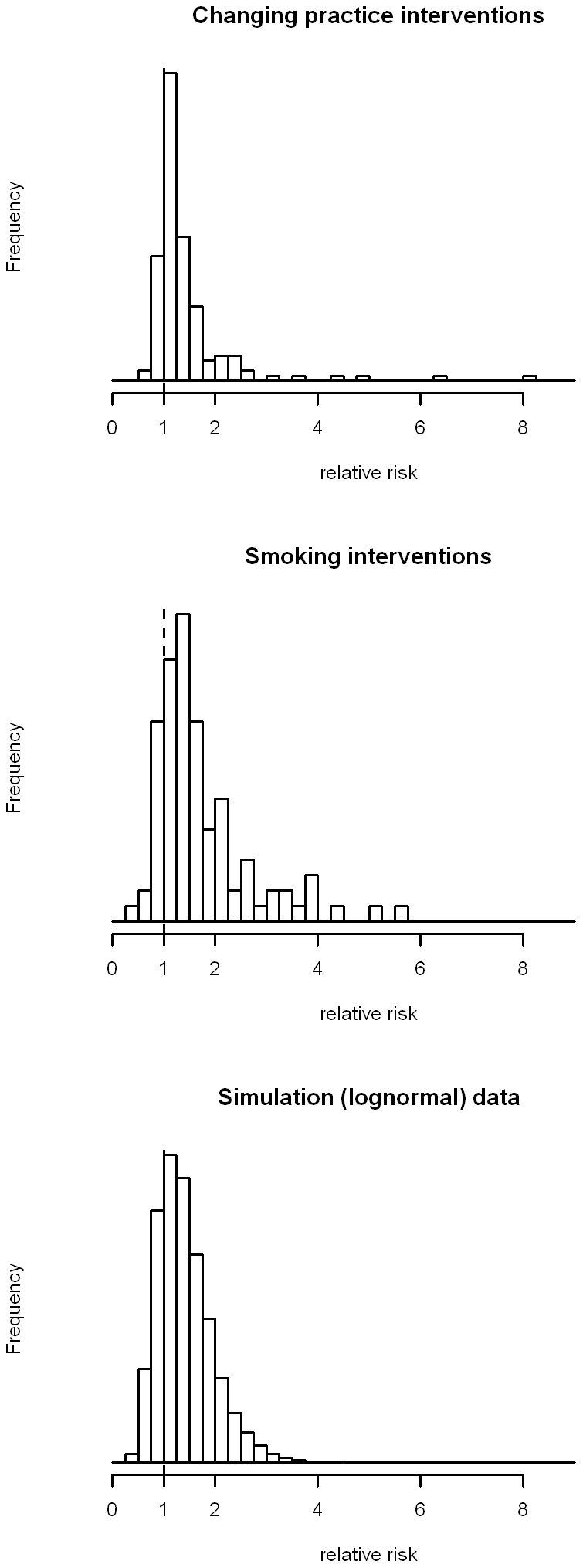
Histograms of pooled effect sizes from three sets of comparisons. (a) shows data from the pooled interventions to change clinical practice (N = 155). (b) shows data from the pooled interventions to help individuals stop smoking (N = 98). (c) shows simulated data from a log-normal distribution with the same log-mean and log-standard deviation as the data in (a).

**Figure 3:**
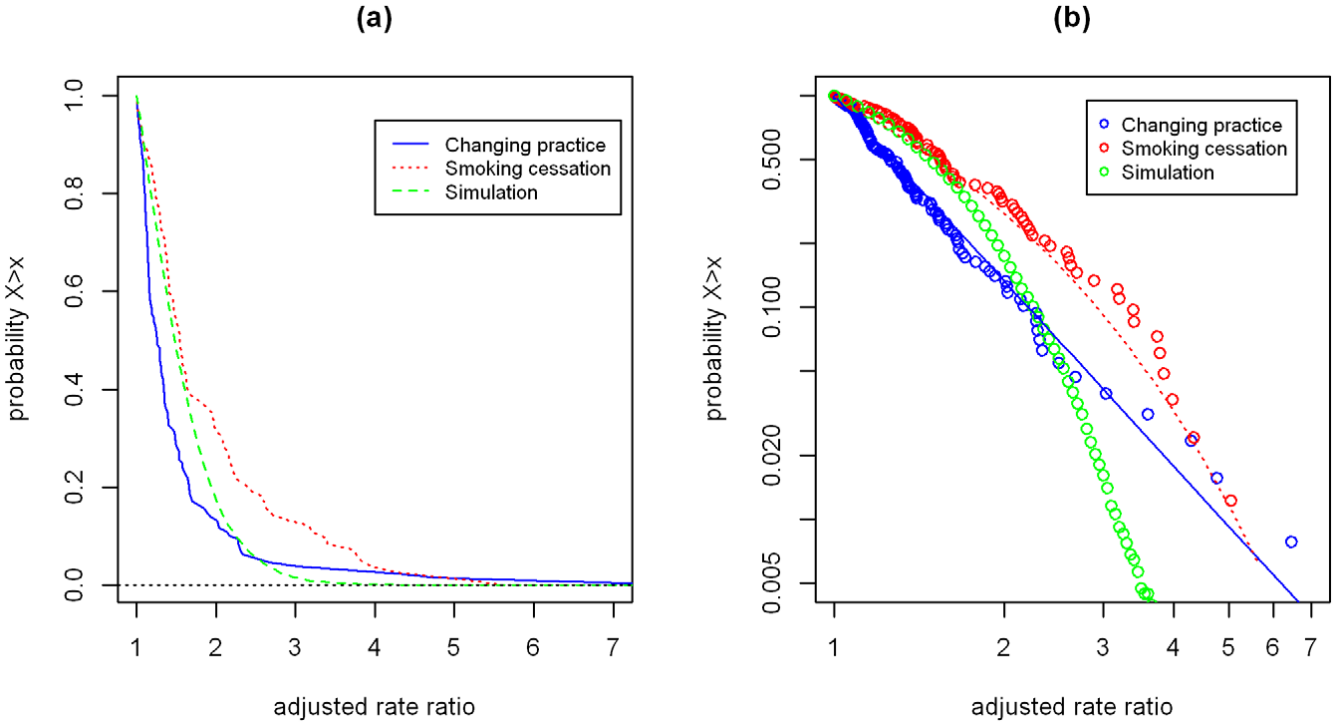
Cumulative distribution of pooled effect sizes. Solid and dashed lines in (b) represent the best fitting inverse power law (changing practice) and stretched exponential (smoking cessation) models identified by maximum likelihood. (a) and (b) show the Log-likelihood of the model fit for inverse power law (a) and stretched exponential (b) distributions. (c) and (d) show the distribution parameters: (c) the exponent of the inverse power law and (d) the shape (solid) and scale (open) of the stretched exponential distribution.

For the changing practice reviews, median relative risk ratio after pooling was 1.17 (before pooling 1.15) with range 0.64 to 8.17. For the smoking reviews, median risk ratio after standardization was 1.42, range 0.47 to 5.62; the simulation data had a median of 1.23 and range 0.23 to 4.65. Twenty seven (17.4%, 95% confidence interval 11.4 to 23.4) risk ratios for the changing practice reviews were less than one, as were 16 (16.3%) for the smoking cessation reviews and 30% of the simulation data points. Histograms of each distribution are shown in figure 2. Figure 3 demonstrates the cumulative density function of the rescaled relative rate ratios for each of the three rescaled distribution on conventional (a) and logarithmic axes (b). These show that both sets of intervention studies possess heavier tails than the log-normal distribution of effect sizes which would be expected by chance. The data for the changing practice interventions appears to fit the inverse power law distribution: of the three sets of data it has the smallest median value and the “heaviest” tail.

### Distribution fitting

The results of maximum likelihood fitting of the EPOC and smoking cessation data to both stretched exponential (Weibull) and inverse power law (Pareto) distributions of the data are shown in table 2. This shows good fit for both the values of relative risk above a threshold of 1 and for absolute values, with the EPOC data fitting the inverse power law distribution better and the smoking cessation fitting the stretched exponential.

**Table 2:** Results of fitting of data to stretched exponential and inverse power law distributions.

Dataset	Distribution	Approach to negative values	N	Exponent	Shape	Scale	Log likelihood	Tests of difference
EPOC	Inverse Power Law	Exclude values<1	128	3.91			−35.3	Inverse power law better fit than stretched exponential p<0.001
		Absolute values	155	4.31			−16.1	
	Stretched exponential	Exclude values<1	128		0.8	0.46	−42.0	
		Absolute values	155		0.77	0.39	−24.5	
Smoking Cessation	Inverse Power Law	Exclude values<1	82	2.79			−80.2	Stretched exponential better fit than inverse power law (excluding values <1) p<0.001; no difference with absolute values (p = 0.77)
		Absolute values	98	3.03			−76.9	
	Stretched exponential	Exclude values<1	82		0.90	0.76	−75.2	
		Absolute values			0.55	0.14	−74.9	

### Sensitivity analysis

The sensitivity analysis showed that resampling with up to 70 additional data points with small effect sizes to simulate publication bias leading to under-reporting of studies with small results increased rather than diminished goodness of fit, as judged by the log-likelihood, with little change in model parameters (data not shown).

## Discussion

We examined the distribution of effect sizes of a range of complex interventions and found heavy tailed distributions typical of those seen in interventions on complex systems. While such distributions are ubiquitous in natural and open systems they have only occasionally been looked for in healthcare [20]; our findings of heavy tails in the effect size distributions of complex interventions support the notion of complex interventions as interventions in complex systems [7].

### Strengths and limitations

A key strength of this study is that it uses data collected and processed by the methodologically rigorous Cochrane review group. This markedly reduces the chance that the distribution is due to the inclusion of methodologically weak studies with high risk of bias. Furthermore, we simulated the effect of publication bias against weak results by adding up to 70 resampled studies with small effect sizes and this did not significantly change our findings. However, the number of suitable reviews was modest. While our criteria were relatively restrictive, we chose to limit ourselves to studies which fitted the models of differing levels of complexity.

The study brought together reviews from different aspects of practice, introducing the possibility of differences between comparisons accounting for our findings. We addressed this by rescaling the effect sizes within each comparison before pooling the data, and inspection of summary measures of the comparisons (table1) suggests that the distributions are broadly similar. While the use of pooled effect sizes in meta-analysis make it possible to compare relatively dissimilar items, they introduce additional potential error. We attempted to reduce this by limiting the analysis to comparisons with 10 or more studies. While the use of relative change values introduced potential bias – studies with smaller baseline values could yield greater relative change for the same absolute change - this was the method used in the Cochrane reviews and so was kept for this analysis.

Twelve studies appeared in two comparisons of changing practice behaviour and six appeared in two comparisons of interventions for stopping smoking. As these resulted in different standardized effect sizes in each comparison we included both instances in the analysis rather than arbitrarily removing one and reducing the sample size. Heavy tailed distributions, such as the inverse power law, typically start at a baseline value of one or zero. Studies with negative effect sizes or fractions of less than one thus present a problem. We took the view that negative effects could arise either through random chance or through interventions leading to change in the unintended direction (so-called unexpected consequences). As the distribution of effects in complex systems relates to the size rather than direction, we deemed it appropriate to take absolute values, however to test for the effects of this we also reported analysis which excluded negative values. Both methods resulted in broadly similar results.

The two distributions tested are not the only heavy tailed distributions and comparable results may have been observed fitting other distributions but we did not test this. As Clauset and Newman [18] argue, the point is less that one specific distribution is correct, rather that a heavy tailed distribution represents a good fit. Our finding that data from the most complex intervention fits best to the inverse power law with the smallest median value and the longest tail, with the moderate complexity intervention fitting a stretched exponential which sits between this and the lognormal distribution of effects which would be expected by chance is in keeping with our model of complexity but requires further testing.

### Comparison with other studies

This is the first study to examine the distribution of effect sizes from complex interventions from the perspective of complex systems. Previous theoretical work has argued that this might be expected [6], [7], [9]. Several authors have argued that the response of theoretical and simulated complex systems to change is inherently unpredictable. These complex systems possess both resilience against change and a capacity to transform in unanticipated ways as local reactions interact with each other and lead to an emergent response. [9] Although heavy-tailed distributions are known to arise in complex systems, the reason for this is not yet clear [30]. Recent work suggests that heavy-tailed distributions may offer an efficient distribution (in information theoretic terms) in respect of members of a group of items, in contrast to a population of individual items [31]. Systems whose group membership follows a heavy tailed distribution may represent an optimal trade-off between robustness and adaptability [32].

### Implications for practice, policy and research

Our findings have implications for the interpretation of intervention studies which go beyond the theoretical importance of considering the complexity involved in so-called complex interventions. These implications relate to the characteristics of the heavy tailed distributions and the inferences which can be made from them.

Each of the reviews included in this analysis reported heterogeneity, in terms of the normal distribution, and none could explain it through meta-regression. Under a heavy-tailed distribution the appearance of a few very large effect sizes is to be expected and the observed values fitted comfortably with this. In practical terms this means that difficult to explain variation may no longer need an explanation, other than that it represents the natural variation of effects seen within a complex system.

There are two additional implication of the heavy tailed distribution for the results of complex interventions. The first arises where policy makers and evaluators seek a grass-roots approach to innovation in multiple sites, with selection of the “best” performer for wider roll-out. This approach runs the real risk of mistaking the random and context-specific effects in a complex system for the inherent merit of the best performing intervention. The second occurs as interventions are reproduced in a range of contexts. As, in a heavy tailed distribution, the vast majority of effects are small, there is the possibility that rolling out apparently successful interventions, may lead to disappointment as smaller effect sizes than originally seen appear more frequently.

### Conclusions

The demonstration of heavy tailed distributions of effect sizes from two types of complex interventions is the first empirical evidence to support the argument that complex interventions represent interventions in complex systems.
